# Identification of *AREB/ABF* Gene Family Involved in the Response of ABA under Salt and Drought Stresses in Jute (*Corchorus olitorius* L.)

**DOI:** 10.3390/plants12051161

**Published:** 2023-03-03

**Authors:** Manuel Sebastian Fiallos-Salguero, Jing Li, Yunqing Li, Jiantang Xu, Pingping Fang, Yankun Wang, Liwu Zhang, Aifen Tao

**Affiliations:** 1 Key Laboratory of Ministry of Education for Genetics, Breeding and Multiple Utilization of Crops, Fujian Agriculture and Forestry University, Fuzhou 350002, China; 2 Fujian Key Laboratory of Crop Breeding for Design, Fujian Agriculture and Forestry University, Fuzhou 350002, China

**Keywords:** *Corchorus olitorius*, *AREB/ABF* genes, genome-wide analysis, abscisic acid, stress response, gene expression

## Abstract

The abscisic acid (ABA)-responsive element binding protein/ABRE-binding factor (AREB/ABF) subfamily members are essential to ABA signaling pathways and plant adaptation to various environmental stresses. Nevertheless, there are no reports on *AREB/ABF* in jute (*Corchorus* L.). Here, eight *AREB/ABF* genes were identified in the *C. olitorius* genome and classified into four groups (A–D) based on their phylogenetic relationships. A *cis*-elements analysis showed that *CoABFs* were widely involved in hormone response elements, followed by light and stress responses. Furthermore, the ABRE response element was involved in four *CoABFs*, playing an essential role in the ABA reaction. A genetic evolutionary analysis indicated that clear purification selection affects jute *CoABFs* and demonstrated that the divergence time was more ancient in cotton than in cacao. A quantitative real-time PCR revealed that the expression levels of *CoABFs* were upregulated and downregulated under ABA treatment, indicating that *CoABF3* and *CoABF7* are positively correlated with ABA concentration. Moreover, *CoABF3* and *CoABF7* were significantly upregulated in response to salt and drought stress, especially with the application of exogenous ABA, which showed higher intensities. These findings provide a complete analysis of the jute *AREB/ABF* gene family, which could be valuable for creating novel jute germplasms with a high resistance to abiotic stresses.

## 1. Introduction

Jute (*Corchorus* L.) is one of the most valuable bast fiber crops in the world. It is composed of two main species: *C. capsularis* (genome size 348 Mb) and *C. olitorius* (genome size 387 Mb) [1], both in the Malvaceae family [2]. The Malvaceae family is considered a large dicot family. It has 4225 diverse species that belong to 244 genera [3]. However, only a few plant species of this family are industrially important crop species, i.e., cotton (*Gossypium*) and jute, which are essential plants for fiber production, and cacao (*Theobroma cacao* L.), the seeds from which are widely used for chocolate production and confectionery [4]. Furthermore, it was found that the *C. olitorius* and *C. capsularis* genomes are highly similar to the *G. raimondii* and *T. cacao* genomes [5,6]. Jute is considered an essential crop with multiple value-added products and uses [7], including industrial applications and social benefits [8]. In recent years, there has been a gradual decrease in the yield and quality of jute products [9] due to unfavorable factors such as climate conditions and contaminated soil and water. Saline and drought conditions are the most severe abiotic factors that limit the development and cultivation of jute [10]. These factors directly affect the physiology of jute through water and ionic stress, ultimately inhibiting leaf expansion [11,12], which significantly reduces the response, adaptability, and assimilation of growth nutrients (e.g., iron and phosphorus). Additionally, abiotic stress has been shown to directly affect lateral root growth, whereas the primary roots are less sensitive to stress conditions, producing the highest toxicity in cells [13].

The resistance of plants to various external stresses can be enhanced by a large set of chemical compounds (i.e., abscisic acid (ABA)) [14]. ABA notably reduces stress damage in plant organs and tissues [15]. A hormone analysis revealed its functions and reactions in vegetative structures [16]. In addition, ABA has many effects on plants. These effects have been identified from plant physical behavior and positive changes in samples treated with exogenous phytohormones [17]. Meanwhile, the biological actions of ABA are exerted through transcription factors (TFs), revealing the signaling pathways of the acid through cells and their functional aspects concerning treatment [18,19]. ABA signals may be expressed via dependent and independent pathways. The ABA-dependent pathway is closely related to the *PYR/CAR* and *PP2C* gene families, which are ABA receptors [20,21,22]. Subsequently, these genes allow SnRK2 to activate ABA-responsive genes such as the abscisic-acid-responsive element binding protein/ABRE-binding factor (*AREB/ABF*) subfamily members [21]. However, the ABA-independent pathway indicates the direct participation of particularly important TFs (e.g., NAC, DREB, and AP2/ERF) [23]. These gene families primarily represent responses to stress through the induction of ABA in plant stress resistance [15,24].

Following the ABA-dependent pathway, interactions involving these large gene families induce the *AREB/ABF* gene subfamily, which activates inducible genes in response to abiotic conditions [25]. *AREB/ABF* subfamily members are a subgroup of the basic leucine zipper (bZIP) TFs, which are the most essential representatives of the ABA-responsive regulatory pathway [26,27]. An analysis of the AREB/ABF *cis*-elements showed a strong relationship with the ABA-responsive element (ABRE; P box) [28], which induced the expression and response of ABA by promoter analysis. Furthermore, the main characteristics of the genes involved were identified through the most significant *cis*-element (i.e., ABRE) in response to ABA. A gene structure analysis revealed a distribution of conserved domains in the C- and N-terminal regions of the sequences [29]. Thus, *cis*-elements and gene structure analyses might regulate the influence and induction of several genes under stressful conditions [30]. In *Arabidopsis*, nine (*ABF1, ABF2, ABF3, ABF4*, *AtDPBF1, AtDPBF2, AtDPBF3*, *AtDPBF4,* and *AtbZIP15*) members of the *AREB/ABF* subfamily possess the bZIP domain in all of their protein sequences [31]. 

Several studies have analyzed and demonstrated the *AREB/ABF* subfamily efficacy in identifying the ABA signaling pathway and improving abiotic stress adaptability and resistance in rice [32], wheat [33], potato [34], sweet potato [35], cotton [36], apple [37], strawberry [38], and rose [39]. To date, there have been few studies in jute regarding AREB/ABF subfamily identification and their gene expression levels under abiotic stress. This study aimed to promote the functional analysis of *AREB/ABF* genes in jute to understand their response to ABA salt and drought stress with the application of exogenous ABA. Moreover, our results could lay the foundation for identifying candidate genes for molecular stress resistance breeding in jute.

## 2. Results

### 2.1. Identification and Characteristics of AREB/ABFs in Jute

*AREB/ABF* subfamily members in the *C. olitorius* genome were identified using Arabidopsis AREB/ABF proteins as a query to search for candidate genes by BLASTP. Eight genes were identified as putative AREB/ABF subfamily members and named *CoABF1* to *CoABF8* based on their chromosome locations. All *CoABF* genes have a bZIP domain, indicating the characteristics of bZIP TFs and a conserved representative of *AREB/ABF* genes. An ExPasy (https://www.expasy.org/, accessed on 11 January 2022) analysis revealed the main differences in the physical and chemical properties of all CoABF proteins. The lengths of all CoABF proteins were predicted to range from 215 aa (CoABF5) to 575 aa (CoABF8) with an average of 373 aa (Table 1). The molecular mass was predicted to range from 23.62 kDa (CoABF5) to 63.11 kDa (CoABF8), and their isoelectric points (pI) ranged from 6.67 (CoABF2) to 9.86 (CoABF1). The subcellular location was evaluated using Cello (http://cello.life.nctu.edu.tw/, accessed on 11 January 2022), predicting that all genes were located in the cell nucleus (Table 1).

### 2.2. Phylogenetic Relationship and Sequence Analysis of AREB/ABFs in C. olitorius

**Figure 1:**
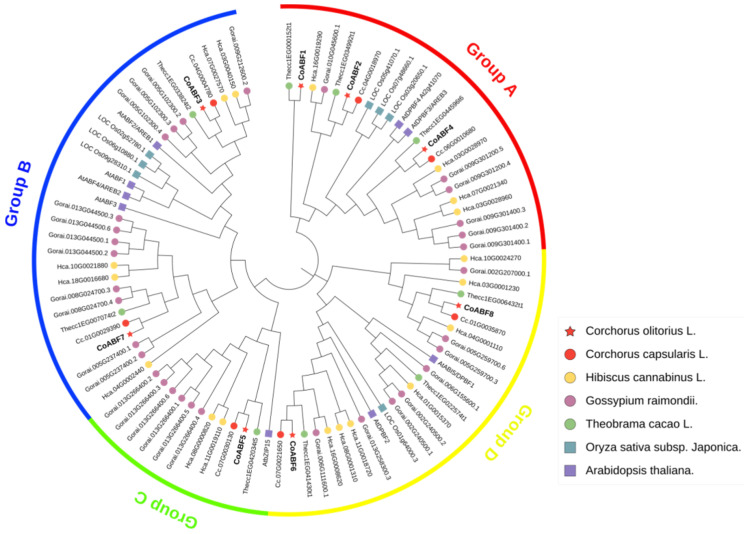
Phylogenetic analysis and family classification of the AREB/ABF proteins were analyzed. The different colored arcs indicate the diverse groups of the AREB/ABF proteins. Protein sequences from jute (*C. olitorius* and *C. capsularis*), kenaf (*H. cannabinus*), cotton (*G. raimondii*), cacao (*T. cacao*), rice (*O. sativa*), and *Arabidopsis* are indicated by red stars, red, yellow, purple, and green circles, and blue and purple squares, respectively.

A non-rooted phylogenetic tree was constructed based on multiple sequence alignments of CoABF proteins with AREB/ABF proteins of *Arabidopsis thaliana* (9), *Oryza sativa* subsp. *Japonica* (7), *Hibiscus cannabinus* (18), *Gossypium raimondii* (32)*, Theobroma cacao* (9), and *Corchorus capsularis* (7) with a highly conserved bZIP TF domain (Figure 1). Ninety AREB/ABF proteins were classified into four groups (A–D) according to the DNA-binding specificity and the expression of *Arabidopsis* AREB/ABF proteins. The largest group was Group A, which contained three CoABF proteins. Groups B and D contained two family members, whereas Group C contained only one. Most CoABF proteins exhibited a close relationship with *C. capsularis* amino acid sequences except CoABF1, which was close to *T. cacao* proteins.

All CoABF proteins were subjected to multiple sequence alignments to analyze their multiple conserved domains. We found that their amino acid structures revealed the basic region of the bZIP domain, in addition to proteins that shared almost the same length among the sequences. The data were collected at a threshold of 50% for conserved sites (Figure S1). An analysis of the CoABF proteins revealed an extremely conserved basic region among the eight members. The distribution of RxxS/T sites among the C1 to C3 domains was conserved in most of the CoABF amino acid sequences. Moreover, except for CoABF5, conserved leucine residues in the bZIP domain were found in their sequences.

### 2.3. Gene Structure and Conserved Motif Analyses of AREB/ABF Family Members in Jute

**Figure 2:**
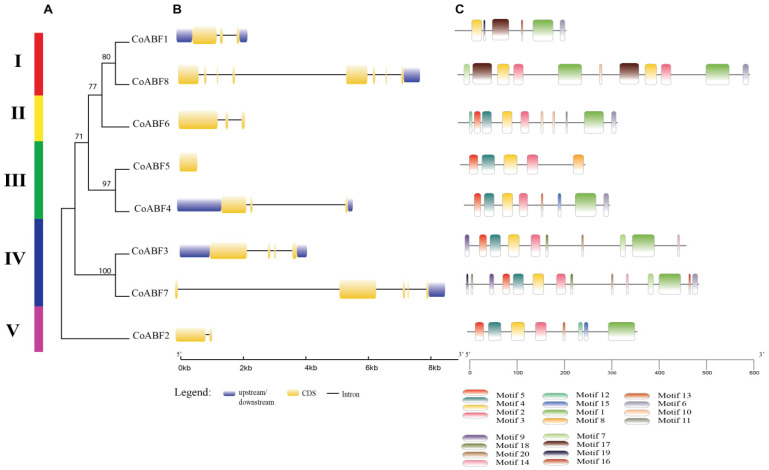
Gene structure and conserved motifs of *CoABF* genes were analyzed. (**A**) A phylogenetic tree was constructed with 1000 bootstraps, using the neighbor-joining method. (**B**) Gene structure was analyzed by Gene Structure Display Server (GSDS 2.0). The untranslated 5′- and 3′- upstream/downstream regions, exons, and introns are represented by blue and yellow boxes and black lines, respectively. (**C**) The conserved motifs were analyzed by the MEME suite web and are displayed in different colors.

To further explore the structure and function of the AREB/ABF family in jute, a sequence domain and motif analyses were performed (Figure 2). As shown in Figure 2A, the *CoABF* genes were classified into five subfamilies (I, II, III, IV, and V) based on the evolutionary tree and conserved motifs. A gene structure analysis revealed the evolution of the gene family based on the distribution of exons and introns among *CoABF* sequences (Figure 2B). A simple distribution of exons and introns was observed among these structures. Our analyses revealed that *CoABF* genes from subfamilies II to V had between one and four introns except for the *CoABF5*, which is an intronless gene. The genes from subfamily I ranged in intron count from two to seven. We found that the least number of exons and introns (2–1) was in *CoABF2*, while the highest number of exons and introns (8–7) was in *CoABF8*. Moreover, the results showed that three *AREB/ABF* (*CoABF1, CoABF4*, and *CoABF6*) members had three exons, and the most extended 5′ UTR region was found in CoABF4.

The conserved motifs predicted for the CoABF proteins were analyzed using phylogenetic tree classification, identifying a similar motif distribution for each subfamily (Figure 2A,C). The positions of the motifs were relatively consistent among subfamilies, especially between subfamilies II and V. Meanwhile, we observed a wide variation in the motif pattern in subfamily I. Our analysis revealed a relationship between motifs 1 and 2 in all CoABF proteins, indicating a highly conserved bZIP domain. Moreover, we observed the presence of motifs 3, 4, and 5 in all amino acid sequences except for CoABF1. These results suggest similar functions of proteins clustered together with identical or similar motif compositions. 

### 2.4. Chromosomal Distribution and Gene Duplication Analysis of CoABF Genes

**Figure 3:**
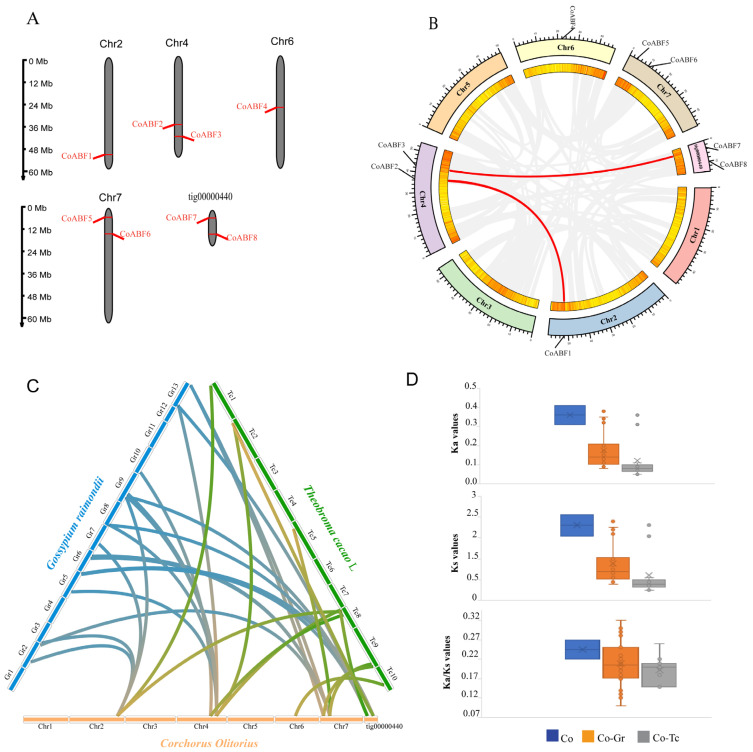
Schematic representations for chromosomal location and synteny analysis analyzed for *CoABF* genes. (**A**) The chromosome (Chr) numbers are exhibited above each chromosome, while the *CoABF* genes are located on the right or left side of the chromosome. The scale bar on the left indicates chromosome length (Mb). (**B**) Segmental duplication of *CoABF* genes in the jute genome was analyzed by gene duplication analysis. Gray lines indicate all synteny blocks in the *C. olitorius* genome and red lines indicate duplicated *CoABF* gene pairs. (**C**) Synteny analysis between *CoABF* genes and *T. cacao* and *G. raimondii* genomes. The orange–green lines indicate the relationship between *C. olitorius* and *T. cacao*, while the orange–blue lines represent the relationship between *C. olitorius* with *G. raimondii.* (**D**) Ka, Ks, and Ka/Ks ratios of duplicated genes. The box plots represent the average and median values of the Ka, Ks, and Ka/Ks values, respectively. Co: * C. olitorius*; Gr: *G. raimondii*; Tc: *T. cacao*.

*CoABF* genes were unevenly distributed across all chromosomes in the jute genome (Figure 3A). *CoABF1* and *CoABF4* are anchored to chromosomes 2 and 6, respectively. Chromosome 4 contained *CoABF2* and *CoABF3*, whereas chromosome 7 contained *CoABF5* and *CoABF6*. *CoABF7* and *CoABF8* were located in a small fragment called tig00000440, corresponding to one of the several fragments found in the jute genome.

In the gene duplication analysis, two gene pairs, *CoABF3*-*CoABF7* and *CoABF1*-*CoABF2*, were classified as segmental duplications, while a tandem duplicated gene pair was not detected (Figure 3B). Additionally, selective pressure was identified using the calculus of the non-synonymous/synonymous (Ka/Ks) ratio, analyzing their role in the expansion of the AREB/ABF gene family. The segmental duplication pairs showed Ka/Ks < 1 ranging from 0.22 to 0.27 and with a mean value of 0.25, indicating a purifying selection in their evolutionary relationship (Figure 3D). The divergence time varied from 47 to 50 Mya, indicating a moderately ancient divergence (Table S1). Furthermore, we analyzed the collinear relationships between the genes from *C. olitorius*, *T. cacao*, and *G. raimondii* (Figure 3C). The results showed a higher number of orthologous gene pairs in *G. raimondii* (20) (Table S2) than in *T. cacao* (11) (Table S3), although most *CoABF* genes were paired with *T. cacao*. The Ka/Ks ratios of the orthologous gene pairs between jute and *G. raimondii* (0.21) and jute and *T. cacao* (0.19) indicated pure selection (Figure 3D). The divergence time was approximately 34 Mya for both relationships.

### 2.5. cis-Element Analysis of AREB/ABF Gene Family in C. olitorius

**Figure 4:**
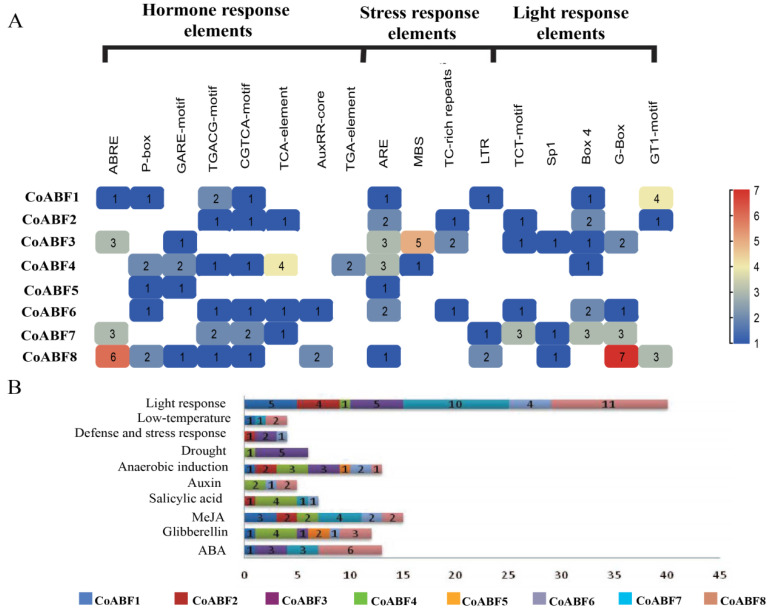
Analysis of *cis*-elements identified in *CoABF* genes. (**A**) Evaluation of *cis*-elements of each *CoABF* gene. (**B**) Analysis of the specific function of *cis*-elements in each *CoABF* gene.

The *cis*-element analysis focused on the promoter regions within 2000 bp upstream of all *CoABF* genes. The results predicted three main *cis*-regulatory element classes: phytohormone, stress, and light responsiveness (Figure 4A). As shown in Figure 4B, the most numerous response elements were found in the promoter region of *CoABFs* related to light response, anaerobic induction, ABA, and MeJA. Thus, four *CoABF* genes (*CoABF1*, *-3*, *-7*, and *-8*) were found with ABRE-responsive elements related to the ABA hormone response. Two genes (*CoABF3* and *CoABF4*) contained drought stress response elements (MBS), and three genes (*CoABF2*, *CoABF3*, and *CoABF6*) were found with defense and stress response elements (TC-rich repeats). Interestingly, only CoABF7 was not found with the anaerobic-induction-responsive element (ARE), while *CoABF3* was widely involved in ABA expression, hormone signaling pathways, and stress responses. These results provide fundamental clues regarding the function of *CoABFs* in response to phytohormones and abiotic stress. 

### 2.6. Analysis of AREB/ABF Protein Network Interactions and Gene Ontology Annotation

Protein interactions involving AREB/ABF members were analyzed using the STRING database (https://string-db.org/, accessed on 15 January 2022) for *A. thaliana* proteins. We observed that AREB3 was homologous to CoABF2, CoABF4, and CoABF5. In addition, a clear relationship (thicker lines) between OST1, SNRK2.4, and DPBF2 (CoABF6) was observed. In contrast, we identified a weak interaction between the CoABF proteins and SNRK2.2 (Figure S2). ABF4 and ABF2 showed homology with CoABF7 and CoABF3, respectively. Our results showed a close relationship with ABI1, which is a key component and repressor of the ABA signaling pathway and maintains the growth stage under normal conditions in the plant [40,41]. All the CoABF proteins were mainly related to bZIP TF and ABA expression at different stages of plant growth. Moreover, the CoABF3 and CoABF7 sequences contained a responsive element (ABRE) in their promoter regions and were related to ABA-inducible genes. 

Furthermore, a gene ontology analysis of the eight CoABF genes demonstrated their principal participation in biological processes, followed by cellular components and molecular functions (Figure S3). The biological processes were the most enriched group, including roles such as cell cycle regulation, DNA replication checkpoints, and ATP biosynthesis. Twenty-one cellular components were enriched, mainly the endomembrane system and intracellular-protein-containing complexes. Finally, ten molecular functions were most relevant to transcription regulatory region sequence-specific DNA binding and double-stranded DNA binding, which were enriched by the transcription of their elements.

### 2.7. Homology Modeling of CoABF Proteins

To better explore their secondary structure and 3D modeling, all CoABF proteins were analyzed concerning their secondary structure using the SOPMA server (https://npsa-prabi.ibcp.fr/NPSA/npsa_sopma.html, accessed on 27 January 2022). The alpha helix content was approximately 28–46%, the extended strand content was 1–12%, the beta-turn content was 0–3%, and the random coil content was 43–61%. The CoABF proteins exhibited all types of secondary structures, and the beta-turn percentages revealed minimal differences among the proteins. As shown in Figure S4, the 3D structural homology modeling of all CoABF proteins was performed using the Phyre2 server (http://www.sbg.bio.ic.ac.uk/~phyre2/html/page.cgi?id=index, accessed on 27 January 2022) with a high confidence and identity percentages of the amino acids. The protein structures of CoABF1–CoABF8 were modeled with 23, 67, 52, 53, 51, 44, 66, and 76 residues, respectively, covering 30–40% of their sequences. Additionally, the CoABF proteins were evaluated with a confidence range of 97–98% for protein structure modeling using the single highest-scoring template. Thus, the prediction of the protein structure of CoABF was highly consistent with the homology modeling, providing essential knowledge regarding their molecular functions.

### 2.8. Expression Pattern of CoABF Genes under ABA Treatment Using Quantitative Real-Time PCR(qRT-PCR)

**Figure 5:**
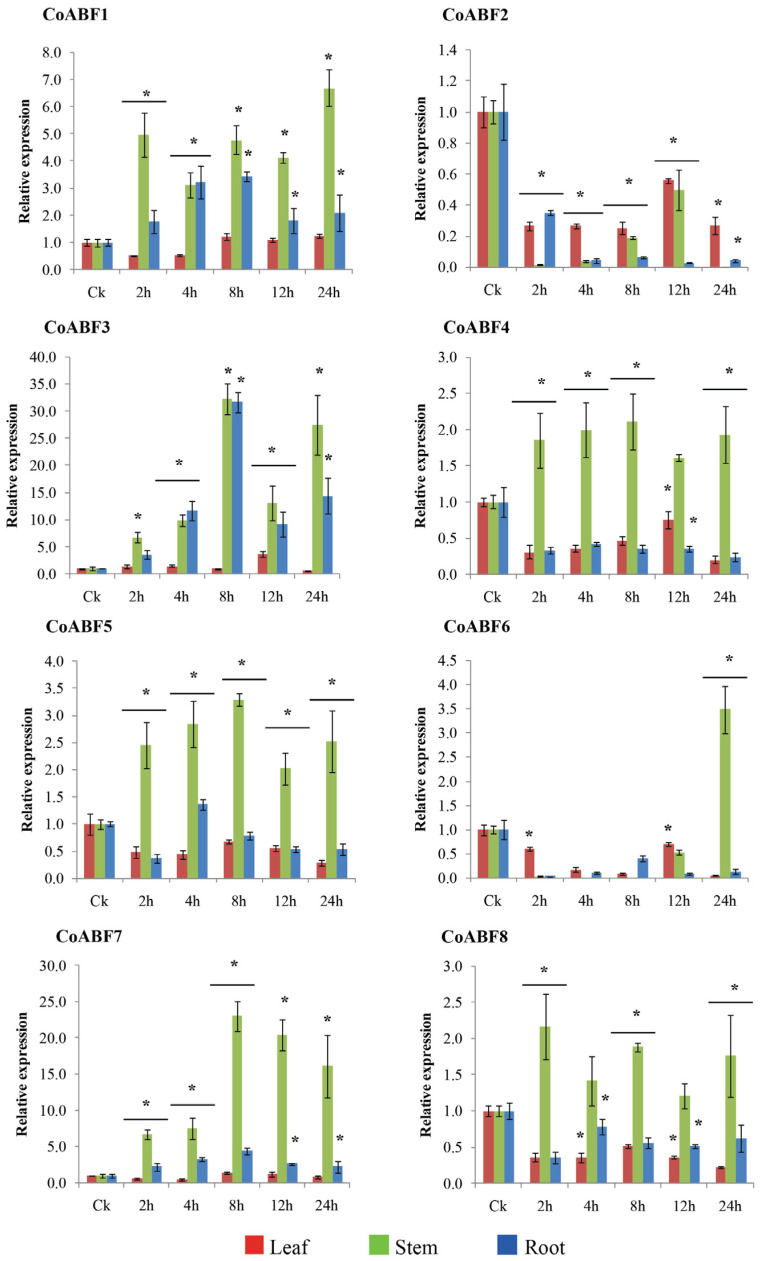
Relative expression of eight *CoABF* genes in jute tissues (leaf, stem, and root) measured under exogenous ABA (100 µmol/L). Data represent the means of three independent replicates ± standard deviation (SD). Asterisks denote statistically significant differences (* *p* ≤ 0.05) compared to the control (CK).

A qRT-PCR analysis was performed to identify the ABA-responsive expression of the jute *AREB/ABF* members. The transcript levels of all *CoABF* genes were evaluated in jute tissues (leaves, stems, and roots) after 100 µmol/L ABA exposure. Most of the *CoABF* genes were upregulated in the stem; nevertheless, their expression was downregulated and upregulated in the leaves and roots (Figure 5) compared to CK expression. For example, *CoABF2* and *CoABF6* demonstrated a negligible ABA response. *CoABF4*, *CoABF5*, and *CoABF8* were only upregulated in the stem, though at a lower intensity compared to the other *CoABF* genes. 

In contrast, *CoABF1* expression was lower during the initial 4 h and then progressively increased in the leaves. Meanwhile, in the stem and root, its expression gradually increased after ABA treatment, reaching maximum levels at 24 h and 8 h, respectively. 

*CoABF3* was highly upregulated in the jute tissues, especially in the stems and roots. We observed that its expression reached its maximum level after 12 h in the leaf, whereas it was significantly induced at 8 h in the stem and root. Interestingly, *CoABF3* peaked at 8 h in both tissues, which was three times higher than that at the other time points. *CoABF7* showed a higher transcript level in the stem than in other tissues, although its maximum level was reached in all tissues after 8 h and then gradually decreased. It is worth noting that *CoABF3* and *CoABF7* were better induced in jute tissues, particularly in the stem by both genes and in the leaf and root by *CoABF3*. Therefore, these two genes showed positive responses at different intensities to ABA signaling in jute.

### 2.9. Expression Levels of CoABF3 and CoABF7 under PEG and PEG+ABA Treatments

**Figure 6:**
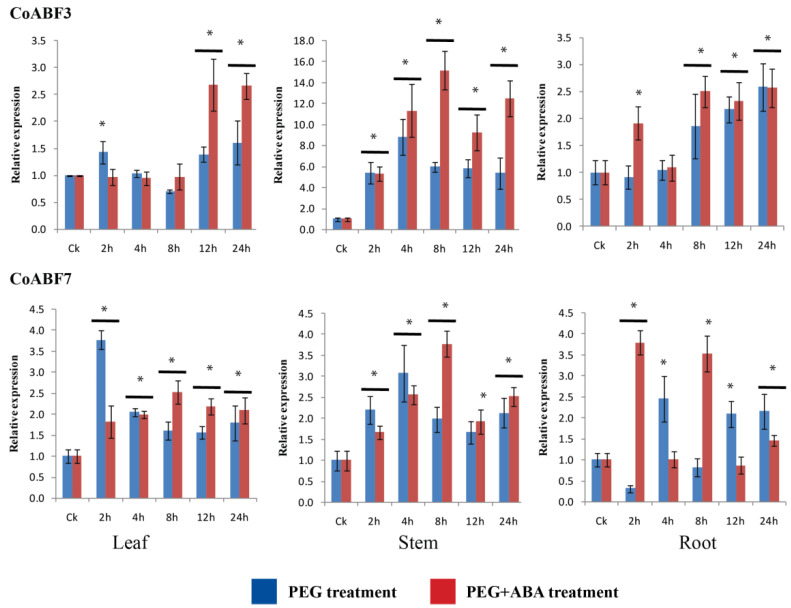
Expression patterns of *CoABF3* and *CoABF7*, measured in jute tissues under PEG (10 mM), and PEG+ABA (10 mM PEG + 25 µmol/L ABA). Data represent the means of three independent replicates ± standard deviation (SD). Asterisks denote statistically significant differences (* *p* ≤ 0.05) compared to the control (CK), respectively.

First, a pre-experiment was carried out to determine the ABA concentration for the drought stress (PEG). The best performance of the jute seedlings was observed with 25 µmol/L ABA among all treatments (10 mM PEG6000 with 0, 10, 25, 50, 75, and 100 µmol/L ABA). Therefore, PEG and PEG-added ABA (25 µmol/L) were used in the drought stress treatment. The results showed that *CoABF3* and *CoABF7* were positively correlated with ABA signaling; therefore, they were selected to measure the ABA response under abiotic (salt and drought) stress in jute. The expression patterns of these genes were measured in jute tissues under PEG and PEG+ABA treatments (Figure 6). Compared to the control, *CoABF3* and *CoABF7* were upregulated and downregulated, respectively, though *CoABF3* was highly upregulated in the stems under PEG+ABA treatment. Following comparative analyses between treatments, *CoABF3* expression showed slight differences within 8 h after treatments; the level was then higher under PEG+ABA than under other treatments in the leaf. In the stem, the gene was strongly induced by the PEG+ABA treatment, revealing a gradual increase within 8 h. The *CoABF3* levels in the roots were slightly different between treatments. In contrast, *CoABF7* was highly expressed during the initial 4 h of the PEG treatment. Subsequently, the expression increased under PEG+ABA in the leaf and stem. In the root, the expression level was more significant at 2 h and 8 h under the PEG+ABA treatment. Interestingly, both genes were activated to a greater extent under PEG+ABA treatment, although *CoABF3* had a more profound response to ABA signaling under drought stress. Furthermore, we observed that both genes responded better with exogenous ABA application than without ABA, even though their intensities were slightly different.

### 2.10. Expression Levels of CoABF3 and CoABF7 under ST and ST+ABA Treatments

**Figure 7:**
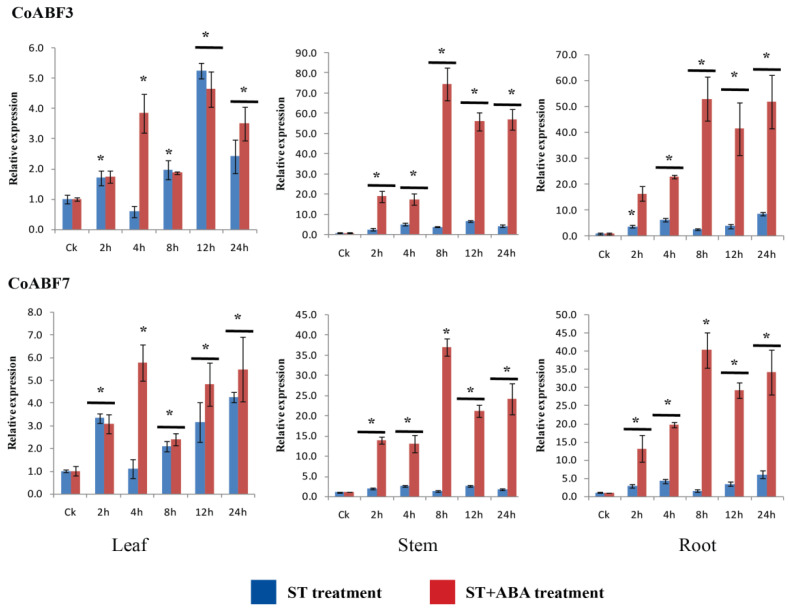
Expression pattern of *CoABF3* and *CoABF7* measured in jute tissues under ST (200 mM) and ST+ABA (200 mM ST + 10 µmol/L ABA). Data represent the means of three independent replicates ± standard deviation (SD). Asterisks denote statistically significant differences (* *p* ≤ 0.05) compared to the control (CK), respectively.

To further investigate the functions of *CoABF* genes in ABA response under salt stress, the expression patterns of *CoABF3* and *CoABF7* in different jute tissues were measured under ST and ST+ABA treatments. The appropriate ABA concentration under salt stress (ST) was determined by a pre-experiment. In all treatments (200 mM NaCl with 0, 10, 25, 50, 75, and 100 µmol/L ABA), we identified the best ABA concentration for salt stress was 10 µmol/L. Therefore, ST+ABA (10 µmol/L) was chosen to identify the expression levels of *CoABF3* and *CoABF7*. As shown in Figure 7, the expression profiles of both genes were mainly activated in the stem and root under the ST+ABA treatment, although the transcription level of *CoABF3* was higher than *CoABF7*. The expression levels of both genes were slightly different between treatments in the leaves; however, at 4 h, their intensities were induced more under ST+ABA than under ST. Compared to the control, both genes were upregulated under both treatments in the stems and roots, even though they were differentially expressed. Notably, both genes reached their maximum levels under ST+ABA treatment at 8 h and then decreased in the stems and roots. It is noteworthy that *CoABF3* and *CoABF7* were significantly induced under both treatments but were higher following ST+ABA treatment, indicating the positive influence of exogenous ABA on the salt stress response. Thus, these genes responded to ABA signaling in jute tissues under salt stress, evaluating their activities with and without ABA.

## 3. Discussion

Jute is referred to as golden fiber because of its color and cost-effectiveness [42], although its marketability has decreased due to the damaging effects of abiotic stress on fiber quality [8,43]. Several studies have shown the importance of essential components in ABA perception and signaling for the stress response in model plants and crops such as *Arabidopsis* [44,45], maize [46], and potato [47]. AREB/ABF gene family members play a crucial role as transcription regulators of ABA gene expression [34] for the adaptation process of plants to external stresses [25,26,48]. Nevertheless, the AREB/ABF gene family has not yet been analyzed in jute. Therefore, this study aimed to identify and express *CoABF* genes that respond to ABA signaling under abiotic stress.

In this study, eight *AREB/ABF* genes were identified in the *C. olitorius* genome, which was classified into four groups (A, B, C, and D). The distribution of chromosomes was partially similar, identifying the undefined location of some *CoABF* genes in the jute genome. This phenomenon might suggest that during its evolutionary process, the jute genome suffered a deviation in the meiotic and mitotic processes, triggering a variation in the overall genome size, ploidy level, and chromosome number or genome fragments and associated diploidization [49,50,51]. Previous studies reported that the number of AREB/ABF members was independent of genome size; for example, nine *AREB/ABFs* were identified in *A. thaliana* [25,52], seven in *S. tuberosum* [34], nine in *P. betulifolia* [39], and fourteen in *P. trichocarpa* [53]. The phylogenetic relationships and conserved motifs of the *CoABFs* were similar in each group, suggesting that these genes might possess similar gene functional sites or participate in the activated bZIP domain [54]. These similar functional sites on genes occur through different physical interactions with DNA [55,56] and the specific binding of TFs to target DNA sequences [26]. These findings indicate that *CoABFs* were attributed to the bZIP domain region due to the phosphorylation of the conserved Ser/Thr residues that regulate the activation of these AREB/ABF members. Pickett and Meeks-Wagner [57] investigated partial redundancy and indicated that distinct roles can be selected for duplicated genes, whereas a shared set of functions is preserved in the same gene family. Nowack et al. [58] reported that genetic redundancy is related to similar positions in two or more genes. However, the inactivation of one of these slightly affects the biological phenotype. In this context, the gene structures and induced expression patterns partially differed among *CoABF* members, indicating that these genes might perform independent functions and functional redundancy.

In addition to evolutionary mechanisms, gene duplication may have provided multiple novel genes with common biological origins during molecular evolution [57,59]. Tandem and segmental duplications led to the divergent expansion of genes in the genome through the generation of gene clusters and homologous genes, respectively [60]. To further confirm this, we found only segmental duplication gene pairs in the jute genome, suggesting that the whole genome of *C. olitorius* may be ancient due to Ka/Ks ratios and divergence time. This is consistent with results reported by Islam [6]. The Ka/Ks ratios of *CoABFs* across Malvaceae genomes indicated that the duplicated genes underwent a purifying selection to remove deleterious variations [61]. Therefore, the close linkages among these crops contributed to the evolutionary analysis of AREB/ABFs in the jute genome.

Protein homology modeling indicated the presence of an alpha helix in all CoABF proteins which was particularly present in the conserved region of bZIP proteins. Similar results have been found in maize [62] and *A. thaliana* [25], which reported the dimerization of proteins before binding to DNA by one amphiphilic alpha-helix. *Cis*-elements play essential roles in the transcription of genes to particular functions, and their divergence is caused by evolutionary changes [63]. In several studies, the effects of plant development may be related to the promoter regions of hormones and stress responses. Our results indicate the formation of transcriptional initiation sites in the promoter regions of *CoABFs*, showing that their transcription can be regulated by light-, hormone-, and stress-responsive genes. Yamaguchi-Shinozaki and Shinozaki [64] confirmed that the transcriptional ignition complex of the core promoter is based on the interaction of transcription factors with *cis*-elements in the promoter region. These findings revealed numerous hormone-responsive elements across the *CoABFs*, followed by light- and stress-responsive elements. Based on this, the hormone signaling pathway might be induced for *CoABF* genes, suggesting a close relationship between hormones. This is consistent with results reported in cassava (*M. esculenta*) in which *MeABFs* revealed putative, *cis*-acting elements related to hormone signaling, stress, light, and the circadian clock [65]. Abscisic acid was found in four *CoABF* genes, indicating the regulation of ABA-dependent gene expression by ABRE-responsive elements [66,67]. Our results are consistent with those of Uno et al. [29] and Choi et al. [55].

Phytohormones play crucial roles in plant growth and in physiological, biochemical, and molecular responses to various environmental stressors [68,69]. In this study, we identified the ABA signaling pathway through the expression patterns of *CoABFs* under exogenous ABA treatment. Among them, *CoABF3* and *CoABF7* were highly sensitive to ABA expression in the stem, followed by roots and leaves. Notably, the highest expression of both genes was reached 8 h after treatment in most tissues. Moreover, the highest response to ABA for both genes was in the stem instead of the root or leaf. Endo et al. analyzed the vascular system in response to stress and found that vascular parenchyma cells are competent to regulate ABA biosynthesis in response to various stresses [70]. North et al. [71] and Osakabe et al. [72] reported that the expression and transport of ABA could be predominantly observed in vascular boundless tissues and other tissues. Based on this, we speculated that ABA is primarily synthesized in the stem and then transported to the target tissues by the xylem and phloem, identifying the transport pathway between the root and shoot of the jute.

To better explore the ABA response, the expression profiles of *CoABF3* and *CoABF7* were evaluated under salt and drought stress with and without exogenous ABA application. Under drought stress, both *CoABF3* and *CoABF7* were upregulated, although *CoABF3* was highly expressed in the stem. Our findings agree with those found in *Arabidopsis* [52], which showed that the water-stress-responsive members of the *AREB/ABF* subfamily regulate the ABRE-dependent ABA signaling involved in drought stress tolerance. Under salt stress, *CoABF3* was significantly induced by exogenous ABA, demonstrating a significant upregulation of the gene involved in stress responses and ABA signaling, especially in the stem and root tissues. Zandkarimi et al. [27] showed that the expression of *AREB1* is involved in high drought and salt stress signal transduction, whereas *AREB2* is induced by salt stress in grapes. This confirms that the application of exogenous ABA showed promising results for causing ABA response in jute under salt stress by increasing the ABA level during the treatment. Several studies have agreed with the use of exogenous ABA to improve the physiological or molecular characteristics of plants [73,74]; however, the ABA concentration can determine its efficacy and applicability. Therefore, our results explain the pivotal role of *CoABFs* in response to the ABA signaling pathway under drought and salt (exogenous ABA) stress, suggesting that they might be involved in the response of jute to abiotic stress.

## 4. Materials and Methods

### 4.1. Sequencing Analysis and Identification of AREB/ABF Gene Family in Jute

Nucleotide and amino acid sequences were analyzed using BioEdit (https://bioedit.software.informer.com/, accessed on 5 January 2022). Protein domains and significant sites were identified using Conserved Domains Database (CDD) tools from the National Center for Biotechnology Information (NCBI). Next, BLASTP and BLASTN searches were performed using NCBI and BioEdit to retrieve the CDS and amino acid sequences of *AtABF1* (At1g49720), *AtABF2/AREB1* (At1g45249), *AtABF3* (At4g34000), *AtABF4/AREB2* (At3g19290), *AtABI5/DPBF1* (At2g36270), *AtDPBF2* (At3g44460), *AtDPBF3/AREB3* (At3g56850), *AtDPBF4* (At2g41070), and *AtbZIP15* (At5g42910) from *A. thaliana* against the *C. olitorius* genome, predicting putative AREB/ABF subfamily members. *AREB/ABF* genes were evaluated with an *E*-value of 1 × 10^−20^ to reduce false positives during their respective BLAST alignments. *C. olitorius* genome files were retrieved from the NCBI database (https://www.ncbi.nlm.nih.gov/, accessed on 5 January 2022), whereas *G. raimondii* and *T. cacao* L genome files were retrieved from the Phytozome database (https://phytozome.jgi.doe.gov/pz/portal.html, accessed on 5 January 2022). The *A. thaliana* genome sequence was obtained from The Arabidopsis Information Resource (TAIR) database (https://www.arabidopsis.org/index.jsp, accessed on 5 January 2022). Amino acid sequence alignments were performed and edited using Clustal X (http://www.clustal.org/, accessed on 6 January 2022) and Jalview (https://www.jalview.org/, accessed on 6 January 2022), respectively. A phylogenetic tree was constructed using the neighbor-joining method with parameters of pairwise deletion and the P-distance model with 1000 replicates in the Molecular Evolutionary Genetics Analysis software MEGA-X package [75]. The results were visualized using the iTOL platform (https://itol.embl.de/, accessed on 7 January 2022).

### 4.2. Protein Characterization and Chromosome Distribution Map of CoABF Genes

Protein characterization was performed using the ExPasyProtParam tool (https://web.expasy.org/protparam/, accessed on 9 January 2022), and the isoelectric point (pI), protein length (aa), and molecular weight of the respective proteins were evaluated. The subcellular location was predicted using the CELLO v.2.5 platforms (http://cello.life.nctu.edu.tw/, accessed on 10 January 2022). Gene positions on the chromosome were analyzed from C. olitorius genome annotation files using MG2C v2.1 (http://mg2c.iask.in/mg2c_v2.1/, accessed on 10 January 2022).

### 4.3. Gene Structure and Conserved Motifs Analysis of CoABF Genes

Both Pfam (https://pfam.xfam.org/, accessed on 11 January 2022) and SMART databases (http://smart.embl-heidelberg.de/smart/set_mode.cgi?NORMAL=1, accessed on 11 January 2022) were used as resources for the identification of non-redundant jute genes. Gene structures were defined using the GSDS 2.0 platform (http://gsds.gao-lab.org/, accessed on 13 January 2022) to identify the number of exons and introns in AREB/ABF genes. Conserved motifs were identified using the MEME Suite database (https://meme-suite.org/meme/, accessed on 14 January 2022), with the following parameters: the maximum number of motifs was 20 and the optimum motif width was 6–50.

### 4.4. Gene Duplication and Ka (Nonsynonymous)/Ks (Synonymous) Ratio Analysis of CoABF Genes

Gene duplication was predicted using BLAST. Orthologous *AREB/ABF* genes were identified in the *C. olitorius* genome, whereas paralogous genes were identified in the *C. olitorius*, *G. Raimondi*, and *T. cacao* L genomes. Tandem and segmental duplications were predicted using the Plant Genome Duplication Database (PGDD) (https://ngdc.cncb.ac.cn/databasecommons/database/id/444, accessed on 20 January 2022). A collinearity analysis was used to analyze the collinearity of *C. olitorius AREB/ABF* genes with the *G. raimondii* and *T. cacao* L. genes using the Multiple Collinearity Scan toolkit (MCSscanX) [76] available in the TBtools toolkit [77]. Simultaneously, the result was obtained from Circos software and the RIdeogram package (https://cran.r-project.org/web/packages/RIdeogram/index.html, accessed on 23 January 2022) (The R Project for Statistical Computing version 4.1.1). The evolution and divergence time of *AREB/ABF* genes were estimated from their CDSs by calculating the Ka (nonsynonymous)/Ks (synonymous) ratio using the DnaSP 6 software (http://www.ub.edu/dnasp/, accessed on 25 January 2022). The divergence time was determined as follows: T = Ks/2 λ × 10^−6^ Mya (where λ = 1.5 × 10^−8^), and the Ka/Ks results reflected the selection pressure in the species [78].

### 4.5. Gene Ontology (GO) Enrichment, Protein Interaction Network, and cis-Elements Analysis of CoABF Genes

GO annotations of *AREB/ABF* genes were retrieved using the Blast2GO software (https://www.blast2go.com/, accessed on 13 January 2022), and their respective analyses were based on biological processes, molecular functions, and cellular components by comparing the reference genome background (*p* < 0.05). GO annotations were visualized using the TBtools software [77]. The protein interaction network was investigated using the STRING 11.0 database (https://string-db.org/, accessed on 15 January 2022). All CoABF amino acid sequences were used as queries, while *A. thaliana* was used as a reference. *cis*-elements were predicted by evaluating 2000 bp upstream regions from the promoter sequences of *AREB/ABF* genes using the PlantCare database (http://bioinformatics.psb.ugent.be/webtools/plantcare/html/, accessed on 17 January 2022). 

### 4.6. Homology Modeling of CoABF Proteins

The three-dimensional structures of the AREB/ABF proteins were determined by searching the CoABF proteins in the Protein Database [79] based on templates with the highest level of similarity. The 3D structure was generated using the Phyre2 server [80] (http://www.sbg.bio.ic.ac.uk/phyre2/html/page.cgi?id=index, accessed on 27 January 2022) by homology modeling in ‘normal’ mode with 98% confidence. Potential active sites of CoABF proteins were predicted using the COACH server (https://zhanggroup.org//COACH/, accessed on 27 January 2022) and displayed using PyMOL (https://pymol.org/2/, accessed on 27 January 2022). A secondary structure analysis of the CoABF proteins was conducted using the SOPMA server (https://npsa-prabi.ibcp.fr/cgi-bin/npsa_automat.pl?page=/NPSA/npsa_sopma.html, accessed on 27 January 2022) [81].

### 4.7. Plant Materials and Growing Conditions

Experiments were conducted using a jute cultivar (Nangyang Changguo) provided by the Laboratory of Genetics and Breeding for Bast Fiber Crops, Fujian Agriculture and Forestry University (Fuzhou 350002, China). The seeds were sown in 12 pots for two weeks, or when approximately three true leaves emerged on each plant. Next, uniform seedlings were transplanted into a hydroponic system with a half-strength Hoagland nutrient solution [82] for 14 days. The solution was replaced with a fresh solution every three days. The seedlings were grown under greenhouse conditions at a temperature of 28–38 °C (night/day), a photoperiod of 14 h, and a relative humidity of 60–80%.

The seedlings were assessed at the six- to seven-leaf stages. The roots, leaves, and stems were harvested under different treatments, including ST and PEG stress, with or without exogenous ABA application. Thus, the jute seedlings’ tissues were subjected to 100 µmol/L ABA [39,83,84], 200 mM NaCl (ST), 200 mM NaCl + 10 µmol/L ABA, 10 mM PEG 6000 (PEG), and 10 mM PEG 6000 + 25 µmol/L ABA, respectively. A quantitative real-time PCR was carried out on the jute tissues (leaves, stems, and roots), which were harvested at 0, 2, 4, 8, 12, and 24 h after treatment (ABA, ST, ST+ABA, PEG, PEG+ABA), snap-frozen in liquid nitrogen, and stored at −80 °C. The experiment included five treatments with three biological replicates.

### 4.8. qRT-PCR Validation

RNA was extracted from approximately 100 mg of fresh leaf, stem, and root tissues using the E. Z. N. A. Plant RNA kit (Omega Bio-Tek, Norcross, GA, USA), according to the manufacturer’s instructions. Subsequently, first-strand cDNA was synthesized from 1 µg of total RNA in a volume of 20 µL using the PrimeScript RT reagent kit (TaKaRa, Kusatsu, Japan) according to the manufacturer’s protocol. The samples were stored at −20 °C. A qRT-PCR was performed to determine the transcriptional expression of these genes. The experiments were conducted using a PCRmax machine EcoRT48 (OSA, London, UK). The qRT-PCR conditions were as follows: 94 °C for 30 s, followed by 40 cycles of 94 °C for 5 s, 60 °C for 15 s, and 72 °C for 10 s. The melting curves of the samples were analyzed. Relative gene expression levels were calculated using the 2^−ΔΔCT^ method [85]. The PCR primers used in this study are listed in Supplementary Table S5.

### 4.9. Statistical Analysis

To validate the reliability of all the samples tested, we analyzed the relative expression of the following reference genes: *PP2A* was used for samples treated with ABA, PEG, and PEG+ABA; *UBC2* for samples treated with ST; and *UBI* for treatment under ST+ABA conditions. Statistical analyses were performed using the statistical software SPSS (version 21.0; SPSS Inc, Chicago, IL, USA) using a one-way analysis of variance. The samples were tested at a 5% significance level and graphs were generated using Microsoft Excel 2007.

## 5. Conclusions

In this study, eight *AREB/ABF* members were identified from the genomic information of *C. olitorius*, which was distributed differently across the chromosomes. An evolutionary phylogenetic analysis classified *CoABFs* into five subfamilies with multiple conserved sites (RxxS/T) and high similarities of domains and motifs in all amino acid sequences. The response elements identified from the *CoABF* promoter regions showed that the hormone- and light-responsive elements were the most common promoters. The analysis of the relationships of *CoABFs* with other Malvaceae family plants revealed their linkages, contributing to the understanding of the evolution of the AREB/ABF gene family in the *C. olitorius* genome through the duplication and functional divergence of these genes. This study focused on the gene expression analysis of the *CoABFs* responsive to ABA under salt and drought stresses and observed that two *CoABFs* (*CoABF3* and *CoABF7*) were significantly related to ABA signaling under ABA treatment. *CoABF3* showed the best expression profile in response to ST and PEG conditions with and without exogenous ABA. The application of exogenous ABA resulted in an increase in ABA levels in jute tissues under salt stress, especially in stems and roots, by *CoABF3* expression. Therefore, our analysis of *CoABF* genes provided a clear understanding of ABA expression and signaling in jute under the stress conditions proposed in this study. Further analysis of these genes could allow for the discovery of their properties and functions under stressful conditions.

## Figures and Tables

**Table 1:** Characteristics of *AREB/ABF* subfamily members in *C. olitorius* genome.

Gene	Gene Id	Theoretical pI	Molecular Mass (kDa)	Protein Length (aa)	Coding Sequence (CDS) Length	Sub-Cellular Location
*CoABF1*	Co.02G0034250	9.86	27.67	253	762	Nuclear
*CoABF2*	Co.04G0023530	6.67	33.45	295	885	Nuclear
*CoABF3*	Co.04G0028660	9.36	49.05	457	1374	Nuclear
*CoABF4*	Co.06G0010990	8.8	35.80	321	966	Nuclear
*CoABF5*	Co.07G0004410	9.52	23.62	215	648	Nuclear
*CoABF6*	Co.07G0011920	9.1	41.88	378	1137	Nuclear
*CoABF7*	Co.v20117110	9.19	53.94	493	1482	Nuclear
*CoABF8*	Co.v20124440	8.48	63.11	575	1728	Nuclear

## Data Availability

Not applicable.

## Supplementary Materials

The following supporting information can be downloaded from: https://www.mdpi.com/article/10.3390/plants12051161/s1. Table S1: Ka/Ks ratio analysis of *AREB/ABF* genes from the jute genome. Table S2: Ka/Ks ratio analysis of *AREB/ABF* genes between jute and cotton genomes. Table S3: Ka/Ks ratio analysis of *AREB/ABF* genes between jute and cacao genomes. Table S4: Secondary structural analysis of CoABF proteins. Table S5: PCR primers for *CoABF* and reference genes. Figure S1: Multiple sequence alignments of CoABF proteins were analyzed. Figure S2: Schematic representations of network interactions of CoABF proteins compared with *A. thaliana* proteins. Figure S3: Gene Ontology of *CoABF* genes was analyzed. Figure S4: Homology modeling of CoABF proteins.
