# Supplementary material for: Identification of AREB/ABF Gene Family Involved in the Response of ABA under Salt and Drought Stresses in Jute (Corchorus olitorius L.)
Source: Plants (Basel). 2023 Mar 3;12(5):1161. doi: 10.3390/plants12051161 (PMC10005393; doi:10.3390/plants12051161)
Supplement: Supplementary file 1 [file plants-12-01161-s001.zip › plants-2242797-supplementary.pdf]

Supplementary materials

Additional File I

Table S1. Ka/Ks ratio analysis of *AREB*/*ABF* genes from the jute genome

Gene ID-1	Gene ID-2	Ka	Ks	Ka/Ks	Selection type	Duplicated type	Mya
CoABF3	*CoABF7*	0.31	1.42	0.22	Purify	Segmental	47.5
CoABF1	*CoABF2*	0.41	1.52	0.27	Purify	Segmental	50.64

Table S2. Ka/Ks ratio analysis of *AREB*/*ABF* genes between jute and cotton genomes

Gene ID-1	Gene ID-2	Ka	Ks	Ka/Ks	Selection type	Duplicated type	Mya
CoABF1	*KJB14378*	0.32	1.96	0.17	Purify	Segmental	65.49
CoABF1	*KJB17601*	0.21	1.02	0.2	Purify	Segmental	33.97
CoABF1	*KJB43284*	0.2	0.67	0.3	Purify	Segmental	22.47
CoABF1	*KJB58061*	0.38	3.03	0.13	Purify	Segmental	100.84
CoABF2	*KJB64369*	0.08	0.83	0.1	Purify	Segmental	27.62
CoABF3	*KJB79321*	0.34	1.83	0.18	Purify	Segmental	60.87
CoABF3	*KJB29467*	0.13	0.53	0.25	Purify	Segmental	17.74
CoABF3	*KJB47401*	0.35	2.44	0.14	Purify	Segmental	81.21
CoABF3	*KJB58506*	0.12	0.49	0.25	Purify	Segmental	16.47
CoABF3	*KJB59845*	0.17	0.55	0.32	Purify	Segmental	18.34
CoABF5	*KJB83823*	0.1	0.81	0.12	Purify	Segmental	27
CoABF6	*KJB83683*	0.15	0.67	0.23	Purify	Segmental	22.19
CoABF6	*KJB16630*	0.13	0.59	0.21	Purify	Segmental	19.77
CoABF6	*KJB35368*	0.11	0.59	0.19	Purify	Segmental	19.73
CoABF6	*KJB36374*	0.1	0.58	0.18	Purify	Segmental	19.36
CoABF7	*KJB47401*	0.09	0.44	0.28	Purify	Segmental	20.1
CoABF7	*KJB32365*	0.11	0.49	0.25	Purify	Segmental	23.6
CoABF7	*KJB79321*	0.15	0.51	0.18	Purify	Segmental	24.7
CoABF7	*KJB58506*	0.18	0.39	0.29	Purify	Segmental	29
CoABF8	*KJB32751*	0.1	0.41	0.22	Purify	Segmental	35.9

Table S3. Ka/Ks ratio analysis of *AREB*/*ABF* genes between jute and cacao genomes

Gene ID-1	Gene ID-2	Ka	Ks	Ka/Ks	Selection type	Duplicated type	Mya
CoABF1	*Thecc1EG000152t1*	0.09	0.45	0.2	Purify	Segmental	15.12
CoABF1	*Thecc1EG034270t1*	0.36	2.48	0.15	Purify	Segmental	82.71
CoABF2	*Thecc1EG034992t1*	0.07	0.48	0.15	Purify	Segmental	16.07
CoABF3	*Thecc1EG007074t1*	0.31	2.12	0.15	Purify	Segmental	70.57
CoABF3	*Thecc1EG033824t2*	0.11	0.42	0.26	Purify	Segmental	13.98
CoABF4	*Thecc1EG044596t4*	0.05	0.24	0.2	Purify	Segmental	7.97
CoABF5	*Thecc1EG042034t1*	0.06	0.32	0.2	Purify	Segmental	10.64

CoABF6	*Thecc1EG022574t1*	0.05	0.23	0.2	Purify	Segmental	7.6
CoABF6	*Thecc1EG041430t1*	0.08	0.39	0.21	Purify	Segmental	13
CoABF7	*Thecc1EG033824t2*	0.09	0.52	0.22	Purify	Segmental	60.57
CoABF8	*Thecc1EG006432t1*	0.07	0.45	0.18	Purify	Segmental	77.24

Table S4. Secondary structure analysis of CoABF proteins

Protein	Alpha helix (%)	Extended strand (%)	Beta turn (%)	Random coil (%)
CoABF1	30.83	8.30	0	60.87
CoABF2	33.22	4.41	1.02	61.36
CoABF3	29.10	12.04	1.97	56.89
CoABF4	34.89	7.79	3.12	54.21
CoABF5	41.86	1.86	2.79	53.49
CoABF6	28.84	8.99	1.59	60.58
CoABF7	33.27	10.75	2.43	53.55
CoABF8	46.43	7.30	3.13	43.13

Table S5. PCR primers for *CoABF* and reference genes

Gene	Type	Sequence	Length	Amplicon
CoABF1	Forward Primer	GGCAAAGACCCACCTACTAATAA	23	97
	Reverse Primer	AGCCTGGACCCGAATTTAAG	20	
CoABF2	Forward Primer	CAGCAGCAGCAAATCACAAT	20	96
	Reverse Primer	CTCTGGAAACCCAACATCCA	20	
CoABF3	Forward Primer	GTTGGTTTAGGAGGAGCAGTC	21	104
	Reverse Primer	GGCGAAACGGAGGAAGTATC	20	
CoABF4	Forward Primer	AAAGGGTGGAGGTTCTGTTG	20	97
	Reverse Primer	CTGATACTGCTGCTGTGGATAC	22	
CoABF5	Forward Primer	GGGAATACAAGAAGGGCAGAA	21	118
	Reverse Primer	AACAGAAAGTCCCGCTTGT	19	
CoABF6	Forward Primer	GCTGAAGCAACTTGTGGTTATG	22	88
	Reverse Primer	CACTGTCCTCCTCAATGTCTTC	22	
CoABF7	Forward Primer	GGGATGGGCATAGTTGGTTTA	21	102
	Reverse Primer	AGGAACTGGTGACAAAGAAGAG	22	
CoABF8	Forward Primer	TGGCGTCGTCTAAGGTGATA	20	85
	Reverse Primer	GTGGAGAGGGAAGAGCATAGA	21	
UBI	Forward Primer	CCACTCTCCACCTTGTCCTC	20	110
	Reverse Primer	CAGCCTCTGAACCTTTCCAG	20	
PP2A	Forward Primer	GCCGACAGATTACTCAAGTG	20	108
	Reverse Primer	GAGAAAGTCCTCCATGCAAA	20	
UBC2	Forward Primer	TATCCGAACAAGCCACCAA	19	112
	Reverse Primer	GTCAGTATTGCAGCTACATCAT	22	

Additional File II

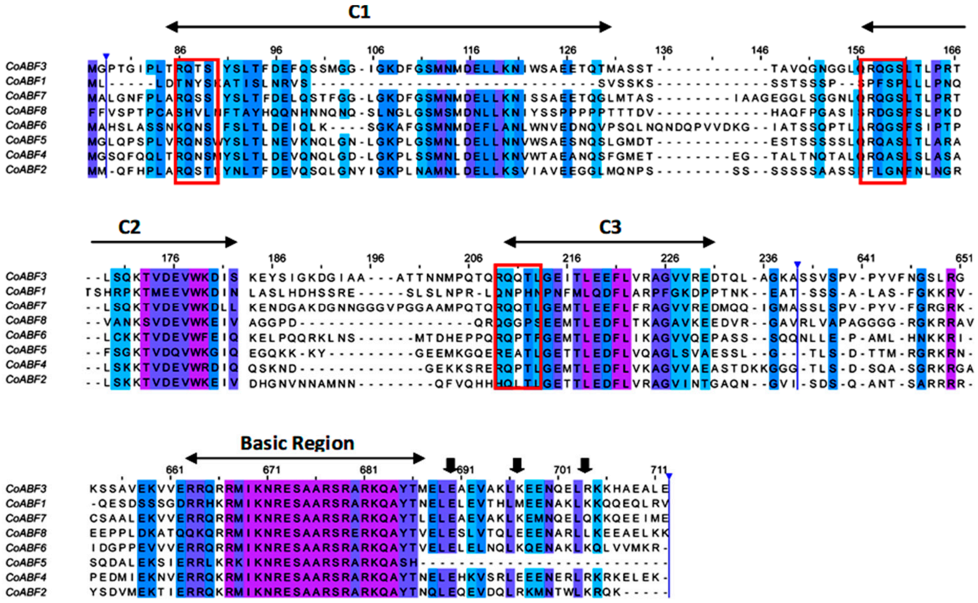

Figure S1. Multiple sequence alignments of CoABF proteins were analyzed. The locations of the C1 to C3 conserved domains and the bZIP domain are indicated by arrows above the protein sequences. Potential phosphor residues (RxxS/T) are identified by a red line. The conserved leucine residues are indicated by a black arrow.

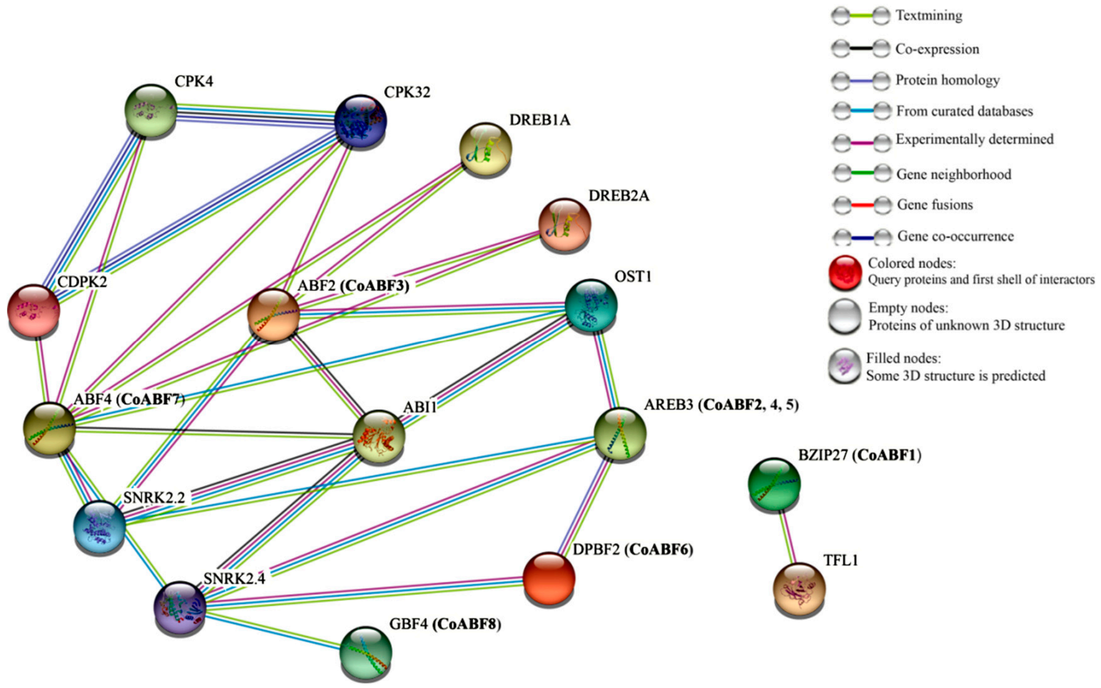

Figure S2. Schematic representations of network interactions of CoABF proteins compared with *A. thaliana* proteins. The different colored lines show the diverse relationships among proteins.

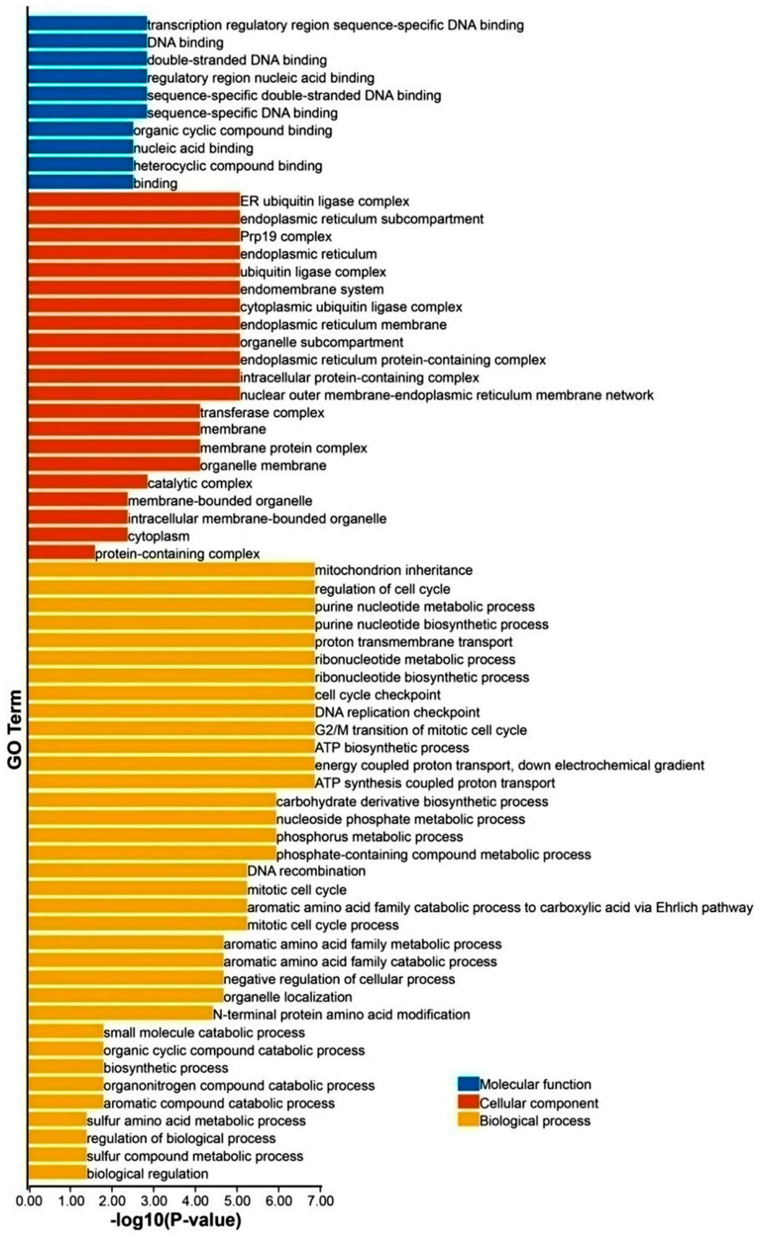

Figure S3. Gene Ontology of *CoABF* genes was analyzed. The molecular function, cellular component, and biological process are indicated by blue, red, and yellow colors, respectively.

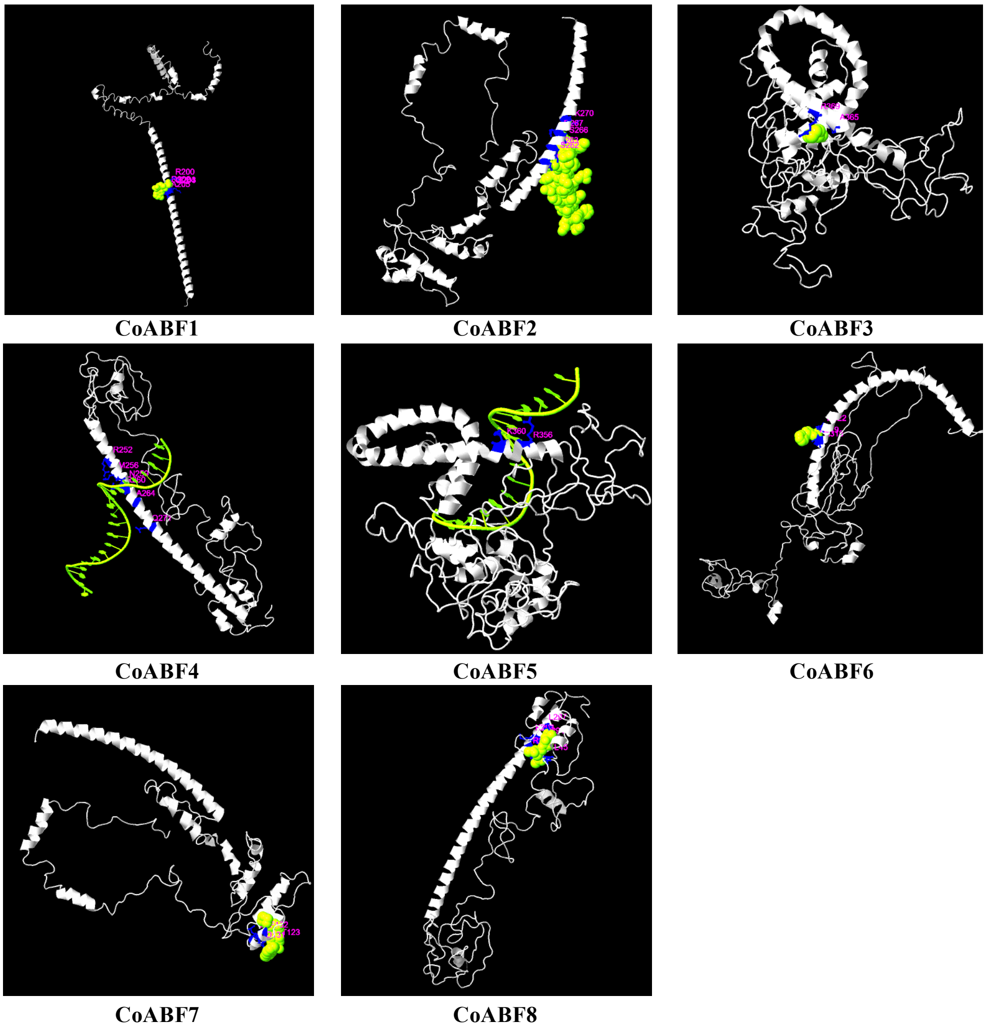

Figure S4. Homology modeling of CoABF proteins. The active sites were predicted by Phyre2with a confidence level >98% and refined by PyMOL.
